# Ipsilateral blooming of microbleeds after Hyperintense Acute Reperfusion Marker sign in an ischemic Stroke patient, a case report

**DOI:** 10.1186/s12883-022-02658-6

**Published:** 2022-04-14

**Authors:** Luigi Francesco Saccaro, Imen Bekri, Maxime De Malherbe, Intissar Hmida, Fernando Pico

**Affiliations:** 1 Versailles Hospital, Neurology and Stroke Care Unit, Le Chesnay, France; 2 Versailles Hospital, Department of Radiology, Le Chesnay, France

**Keywords:** Hyperintense Acute Reperfusion Marker, Cerebral microbleeds, Subarachnoid hemorrhage, Cerebrovascular disease/Stroke, FLAIR hyperintense cerebrospinal fluid, Brain-blood-barrier disruption, Case report, Magnetic resonance imaging (MRI), Atrial fibrillation, Ischemic stroke, Cerebrovascular disease/Stroke, Blood-brain barrier

## Abstract

**Background:**

Hyperintense Acute Reperfusion Marker (HARM) is a hyperintense subarachnoid signal on FLAIR MRI sequence caused by gadolinium contrast leakage into the subpial space. While, on FLAIR, HARM may mimic subarachnoid hemorrhage, it is differentiated from it on computed tomography (CT) and SWAN MRI sequences. Cerebral microbleeds are black, rounded spots on SWAN caused by blood-products deposition following red blood cell leakage from small cerebral vessels brain. Both microbleeds and HARM carry important prognostic implication as they are associated with blood-brain barrier disruption and an increased risk of intracerebral hemorrhage.

**Case presentation:**

A 79-year-old man presented with aphasia and right hemiparesis due to ischemic stroke with left middle cerebral artery occlusion. Admission NIHSS score was 7, and he was successfully treated by intravenous thrombolysis and mechanical thrombectomy. On day 1, his clinical condition worsened, and he had an urgent gadolinium-enhanced MRI. There was no evidence of early recurrence, nor of hemorrhage on SWAN or on FLAIR. Left middle cerebral artery was permeable. The patient was anticoagulated for newly diagnosed atrial fibrillation, and a second MRI following a generalized tonic-clonic seizure showed multiple left hemispheric diffusion-weighted imaging (DWI) hyperintense spots and a left hemispheric sub-arachnoid hyperintensity on FLAIR, compatible with a subarachnoid hemorrhage. This diagnosis was excluded by SWAN MRI sequence and a normal cerebral CT the same day. The diagnosis of HARM was retained. At day 9, patient’s condition improved, and a control MRI did not show evidence of HARM. However, numerous microbleeds were detected in the left hemisphere only (ipsilateral with HARM and stroke).

**Conclusions:**

This case highlights first of all the importance of differentiating HARM and subarachnoid hemorrhage, especially in an anticoagulated patient with clinical aggravation. Secondly, it is crucial to identify microbleeds and understand their pathophysiology, as they are associated with higher risk of hemorrhage and stroke recurrence in ischemic stroke patients. Finally, the mono-hemispheric appearance of microbleeds in this case suggests for the first time that, in some acute ischemic stroke patients, a relationship between HARM and cerebral microbleeds may exist.

## Background

The HARM (Hyperintense Acute Reperfusion Marker) sign is defined as a hyperintense subarachnoid signal on FLAIR [1–3] post-gadolinium-contrast administration*.* It has been attributed to gadolinium contrast leakage into the subpial space due to BBB disruption caused by brain lesions such as ischemic stroke [4–6]. Brain ischemia depletes cellular adenosine triphosphate (ATP), leading to impairment of the sodium-potassium-ATPase. This increases intracellular potassium, lactic acidosis, and extracellular glutamate release [7]. These alterations favor extracellular matrix degradation, as well as tight junctions and BBB disruption, which may be exacerbated if post-ischemic reperfusion takes place. In such a case, first, there is a hyperemic phase that increases BBB permeability. Immediately after this, swelling of endothelial cells and microvascular obstruction lead to a so-called “no-reflow effect” and to a hypoperfusion stage [7]. The ensuing brain tissue nutritional deficiency enhances inflammation and oxidative stress (exacerbated by the partially restored oxygen supply), further disrupting BBB integrity [7]. The resulting increased paracellular permeability can be attributed to a biphasic response. At first, 3–8 h after reperfusion, BBB disruption is mainly due to the aforementioned degradation of the extracellular matrix, inflammation, and oxidative stress. Then, 18–96 h after reperfusion, BBB alterations are associated with vasogenic edema and angiogenesis, which further increase BBB permeability to macromolecules [7]. These changes not only increase the risk of hemorrhagic transformation but also facilitate gadolinium contrast leakage in the subpial space, thus leading to the HARM sign. This cerebrospinal fluid signal hyperintensity has been reported in prospective studies to appear both close to or far from ischemic lesions [8, 9], and it usually lasts from about 3 h to 2 days after injection with gadolinium but, in some cases, HARM persists for up to 6 days [4, 5, 10]. It can be identified in a variety of conditions presenting BBB disruption, including seizures, intracerebral hemorrhages, transient ischemic attacks, and systemic inflammation [2, 4, 10–13]. Cerebral microbleeds are millimetric (< 10 mm) ovoid or rounded spots of hypointense (black) signal drop on SWAN. Like HARM, microbleeds are an MRI finding that should not be neglected, since they are a risk factor for intracerebral hemorrhage (particularly in anticoagulated patients), as well as for ischemic stroke recurrence in patients with brain ischemia [14]. Again, like HARM, cerebral microbleeds are believed to be associated with BBB disruption, which leads to leakage from capillaries and arterioles of red blood cells. These are phagocyted and degraded by microglial cells, leading to the formation and accumulation of iron products (e.g. hemosiderin). Hemosiderin-laden microglia usually remain in the same cerebral region for the patient’s lifetime, typically next to arterioles affected by microangiopathies [15]. Therefore, SWAN allows the detection of hemorrhages that may have occurred at any time in the patient’s life. This MRI sequence is usually chosen since it magnifies microbleeds due to the blooming artifact caused by paramagnetic substances, like blood-derived hemosiderin [14]. Mixed (i.e. lobar and subcortical) patterns of cerebral microbleeds distribution suggest mixed etiologies, while exclusively lobar microbleeds probably indicate cerebral amyloid angiopathy, and subcortical ones are suggestive of hypertension and/or arteriolosclerosis [14, 15].

This case report highlights first of all the importance of differentiating HARM and subarachnoid hemorrhage. Secondly, it shows an example and timing of the appearance of microbleeds and discusses their pathophysiology. Finally, for the first time, this case report suggests that, in some acute ischemic stroke patients, a relationship between HARM and cerebral microbleeds may exist.

## Case presentation

**Fig. 1:**
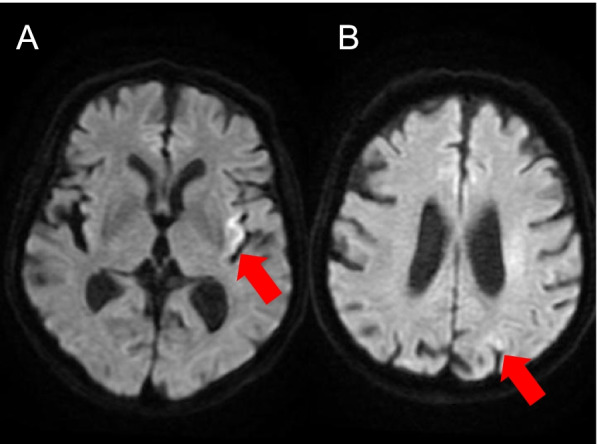
Day 0 DWI. **A** and **B** Initial acute ischemic stroke (red arrows) on DWI sequence at day 0. DWI indicates diffusion-weighted imaging

**Fig. 2:**
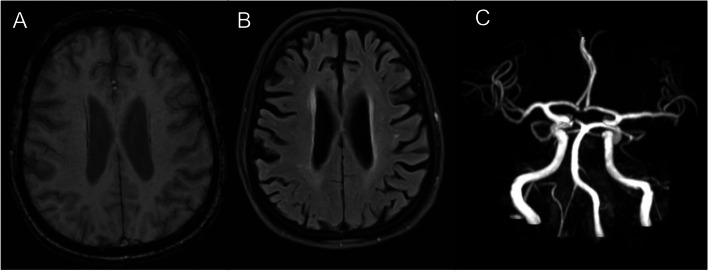
Day 1: post-Thrombectomy Recanalization of the Left Middle Cerebral Artery without Evidence of Hemorrhage or the HARM Sign. **A** SWAN MRI sequence shows no evidence of post-thrombectomy hemorrhage. **B** Post-thrombectomy FLAIR MRI sequence shows no evidence of the HARM sign. **C** 3D time of flight MRI sequence shows post-thrombectomy TICI 3 recanalization of the left middle cerebral artery. FLAIR indicates fluid attenuated inversion recovery; HARM, hyperintense acute reperfusion marker; MRI, magnetic resonance imaging; SWAN, susceptibility-weighted angiography; TICI, Thrombolysis In Cerebral Infarction

**Fig. 3:**
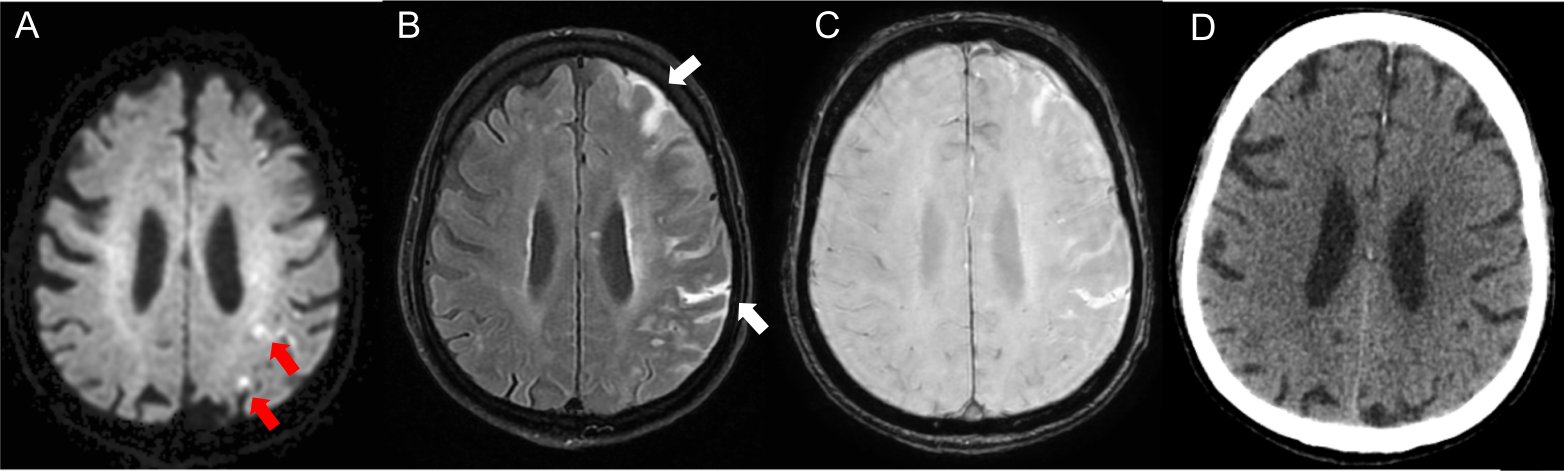
Day 2: Hyperintense Acute Reperfusion Marker Sign Associated with Acute Ischemic Lesions, Without Evidence of Subarachnoid Hemorrhage. **A** DWI sequence shows recent ischemic lesions (red arrows). **B** Non-enhanced FLAIR MRI sequence shows left hemispheric sub-arachnoid hyperintensity (white arrows). **C** SWAN MRI sequence shows no evidence of hemorrhage. **D** Brain CT shows no evidence of hemorrhage. DWI indicates diffusion-weighted imaging; FLAIR, fluid attenuated inversion recovery; MRI, magnetic resonance imaging; SWAN, susceptibility-weighted angiography

A 79-year-old Caucasian, right-handed man without notable clinical history, excluding daily smoking of cigars and occasional alcohol consumption, presented to the emergency department with aphasia and right hemiparesis due to an ischemic stroke (Fig. 1) with proximal left middle cerebral artery occlusion (day 0). The rest of the clinical examination was unremarkable. Admission National Institutes of Health Stroke Scale (NIHSS) score was 7, and he was treated by intravenous thrombolysis (3.5 h after symptom onset) and two-pass mechanical thrombectomy (at 5.5 h) with Thrombolysis in Cerebral Infarction 3 recanalization and stability of the neurological deficits. On day 1, his clinical condition worsened (NIHSS score 11), with mutism, right facial palsy, dysphagia, right hemianopsia, and right arm paralysis and he had an urgent gadolinium-enhanced magnetic resonance imaging (MRI) to exclude early recurrence, hemorrhagic transformation and arterial reocclusion. There was no evidence of early recurrence, nor of hemorrhage on the susceptibility-weighted angiography (SWAN) (Fig. 2A) or on non-enhanced fluid attenuated inversion recovery (FLAIR) (Fig. 2B) MRI sequence. Left middle cerebral artery was permeable (Fig. 2C). For newly diagnosed atrial fibrillation, enoxaparin was started after the aforementioned MRI on day 1 at 60 mg twice daily, which was switched to apixaban 5 mg twice daily on day 6. On day 2, he had a generalized tonic-clonic seizure. Urgent MRI showed multiple left hemispheric diffusion-weighted imaging (DWI) hyperintense spots (Fig. 3A) and a left hemispheric sub-arachnoid hyperintensity on FLAIR (Fig. 3B), which was not present on the previous FLAIR images (Fig. 2B) and was compatible with a subarachnoid hemorrhage. This diagnosis was excluded by SWAN MRI sequence (Fig. 3C) on the same MRI and a normal cerebral computed tomography (CT) (Fig. 3D) the same day. The diagnosis of Hyperintense Acute Reperfusion Marker (HARM) due to extravasation of gadolinium into the cerebrospinal fluid, explained by brain-blood barrier (BBB) disruption [1, 2] was retained. An EEG on day 2 did not show epileptic abnormalities. Supra-aortic trunk echo-Doppler showed small, non-stenosing bilateral plaques of the internal carotid arteries.

**Fig. 4:**
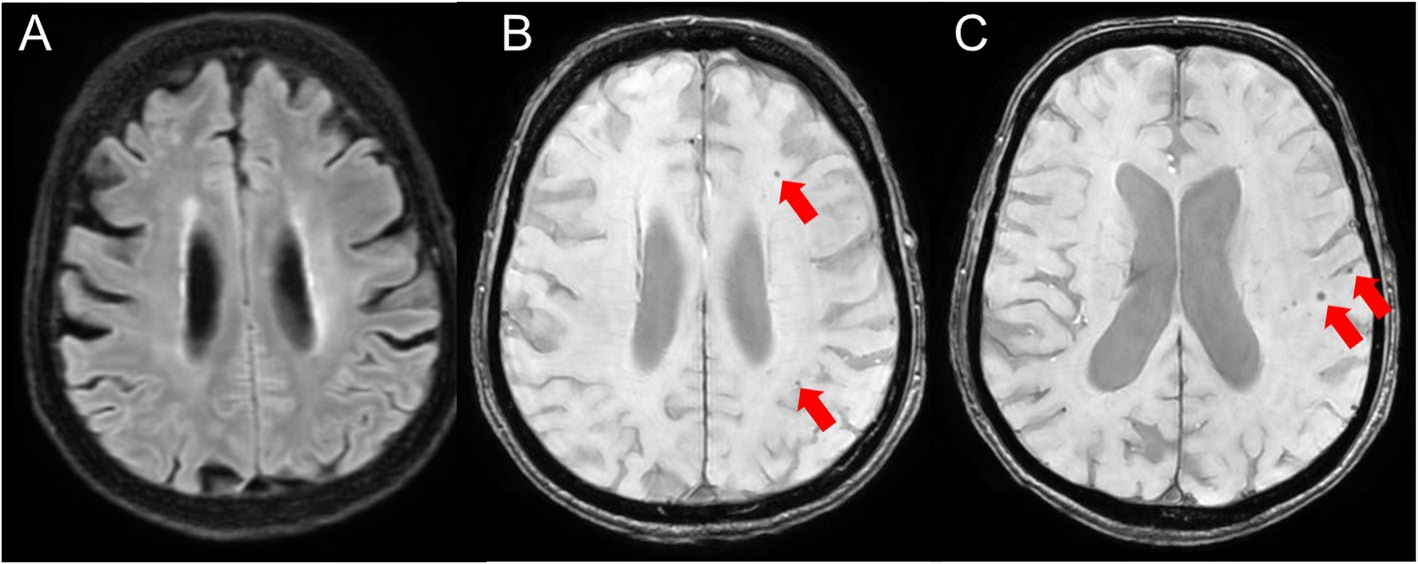
Day 9: blooming of Microbleeds and Disappearance of the HARM Sign. **A** Non-enhanced FLAIR MRI sequence shows no more evidence of the HARM sign 7 days after the acquisition of the images depicted in Fig. 2. **B** and **C** SWAN MRI sequence shows the appearance of multiple hemosiderin spots suggestive of microbleeds (red arrows). FLAIR indicates fluid attenuated inversion recovery; HARM, hyperintense acute reperfusion marker; MRI, magnetic resonance imaging; SWAN, susceptibility-weighted angiography

The patient’s condition improved, with an NIHSS score of 4 on day 9 (persistence of aphasia, mild gait instability, and facial paralysis). Day 9 MRI (Fig. 4) did not show evidence of HARM. However, 28 diffuse dot-like hemosiderin depositions compatible with numerous microbleeds were detected by SWAN in the left hemisphere only (ipsilateral with HARM and stroke). Only two of these microbleeds appeared where a DWI hyperintensity had previously been present. Blood tests showed high C-reactive protein (20 mg/L) and creatinine (114 μmol/L), although he had no history of chronic kidney disease and no clinical arguments for an infective endocarditis, which was excluded with thanks to normal transthoracic and transesophageal echocardiography and two negative blood cultures. Despite the increased risk of intracerebral hemorrhage associated with the appearance of microbleeds, we did not interrupt apixaban treatment due to the high risk of recurrent ischemic stroke in this patient with symptomatic atrial fibrillation, and due to and the very low DWI volumes of the ischemic lesions (< 3 cc). This decision was taken after a multidisciplinary discussion with the cardiologists.

## Discussion and conclusions

As described in the Background, correct identification of HARM has important clinical implications, firstly to differentiate it from a subarachnoid hemorrhage, especially in association with clinical aggravation in an anticoagulated patient. While a subarachnoid hemorrhage appears as a hyperintense subarachnoid signal on FLAIR, like HARM, it is also hyperdense on CT and hypointense on SWAN, contrary to HARM, which is not usually detected on CT (Fig. 3D) (although a recent case report argues that in some cases this might possible [16]) or SWAN (Fig. 3C). Secondly, HARM has been linked with worse clinical outcome [17] and post-ischemic stroke hemorrhagic transformation [1, 6] and could thus help to identify high-risk patients. While its incidence is variable depending on the population studied, HARM may be identified in up to 33–51% of ischemic stroke patients after gadolinium administration [3, 6].

**Fig. 5:**
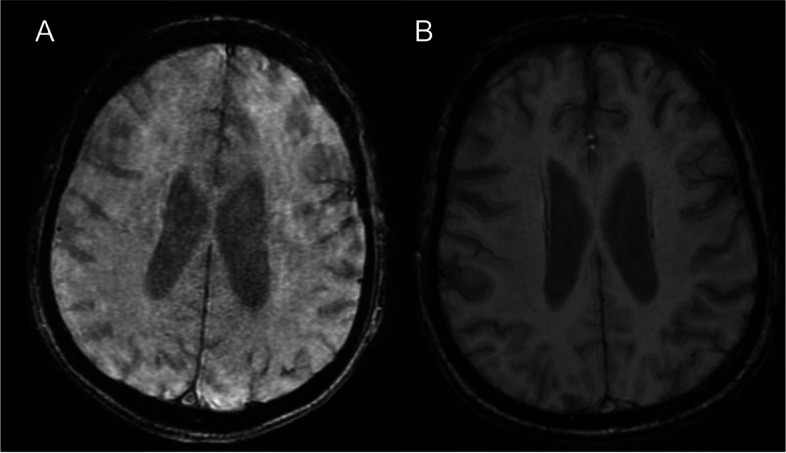
Comparison between SWAN MRI sequences before and after early neurological deterioration. **A** Day 0 SWAN MRI before early neurological deterioration. **B** Day 1 SWAN MRI after early neurological deterioration

Our patient had numerous characteristics that have been associated with HARM in the existing literature, i.e., small recent ischemic strokes of likely cardioembolic etiology, reperfusion after thrombolysis and multiple-pass thrombectomy, clinical aggravation, advanced age, acutely reduced kidney function, biological inflammation, and an epileptic seizure [1–3, 8, 12, 18, 19]. Interestingly, we did not identify gadolinium leakage in ocular structures in this patient, which has also been associated with BBB disruption and ischemic stroke [20, 21]. Of note, the cause of early neurological deterioration in this patient might be explained by the new ischemic strokes found on Day 2 DWI (Fig. 3A) and/or by the generalized tonic-clonic seizure. In fact, SWAN on Day 1 did not show hemorrhagic transformation, as compared to Day 0 SWAN (Fig. 5).

In our patient, we performed a control MRI 7 days after detection of HARM, which showed complete regression of the hyperintense subarachnoid signal on FLAIR (Fig. 4A). On the SWAN sequence of this MRI, we observed the unexpected appearance of multiple microbleeds (Fig. 4B, C). In a recent pooled analysis of 38 cohorts, cerebral microbleeds were found in 28% of the 20,322 ischemic stroke or transient ischemic attack patients studied [14]. In our case, microbleeds appeared exclusively in the same hemisphere as the HARM sign. We exclude that our patient’s microbleeds could be caused by amyloid angiopathy or other systemic diseases, given their unilateral nature and the fact that they were not present on the initial MRI. Anticoagulation use has been associated with the appearance of microbleeds [22], although a recent prospective study suggests that this association is weaker in patients taking direct oral anticoagulants, as in our case, than in those treated with Warfarin [23]. While we cannot rule out an additive effect of anticoagulation in the blooming of unilateral microbleeds in our patient, localization and timing suggest a close relationship between recent ischemic stroke, HARM, and cerebral microbleeds in this case, and it could be speculated that the increased risk of hemorrhagic transformation associated with HARM may be mediated by microbleeds appearances.

To sum up, not only does this case highlight the importance of differentiating HARM and subarachnoid hemorrhage, and of identifying microbleeds, but it also suggests for the first time that, in some acute ischemic stroke patients, a close relationship between HARM and cerebral microbleeds may exist, which may be explained by the importance of BBB disruption in their pathophysiology.

## Data Availability

Data sharing is not applicable to this article as no datasets were generated or analyzed during the current study. However, clinical raw data will be provided upon request to the corresponding author, Dr. Luigi Francesco Saccaro (email address: luigisaccaro@gmail.com).

## Abbreviations

ATP: Adenosine triphosphate
BBB: Brain-blood barrier
CT: Computer tomography
DWI: Diffusion-weighted imaging
FLAIR: Fluid attenuated inversion recovery
HARM: Hyperintense acute reperfusion marker
MRI: Magnetic resonance imaging
NIHSS: National Institutes of Health Stroke Scale
SWAN: Susceptibility-weighted angiography
